# Deep Trichophytosis Mimiking Superinfected Bacterial Folliculitis

**DOI:** 10.1007/s11046-024-00872-z

**Published:** 2024-07-05

**Authors:** Lina Weiss, Sara E. Cerminara, Riccardo Curatolo, Hazem A. Juratli, Matiar Madanchi

**Affiliations:** 1 Department of Dermatology, University Hospital Basel, Burgfelderstrasse 101, 4055 Basel, Switzerland; 2 Department of Dermatology, Dermatopathology Training Center, University Hospital Basel, Basel, Switzerland; 3 Institute of Medical Genetics and Pathology, University Hospital Basel, Basel, Switzerland

A 33-year-old healthy male presented to our dermatology department complaining of painful skin changes on the lower left abdomen and left thigh. The patient reported noticing the appearance of these lesions approximately for 3 months, which had progressively worsened over time with a significant increase in pain and size. He denies any fever or other symptoms besides the pain, as well as recent travel or unprotected sexual intercourse. The patient stated that his family physician initially suspected an eczema and therefore initiated treatment with ultra-high potency corticosteroids (clobetasol propionate) for a total of 2 weeks without improvement. Subsequently, the family physician suspected a bacterial infection and prescribed systemic antibiotic therapy with amoxicillin/clavulanic acid 1000 mg twice daily for 10 days, combined with topical clotrimazole, also with no benefit, but showing progression of the lesions.

**Fig. 1:**
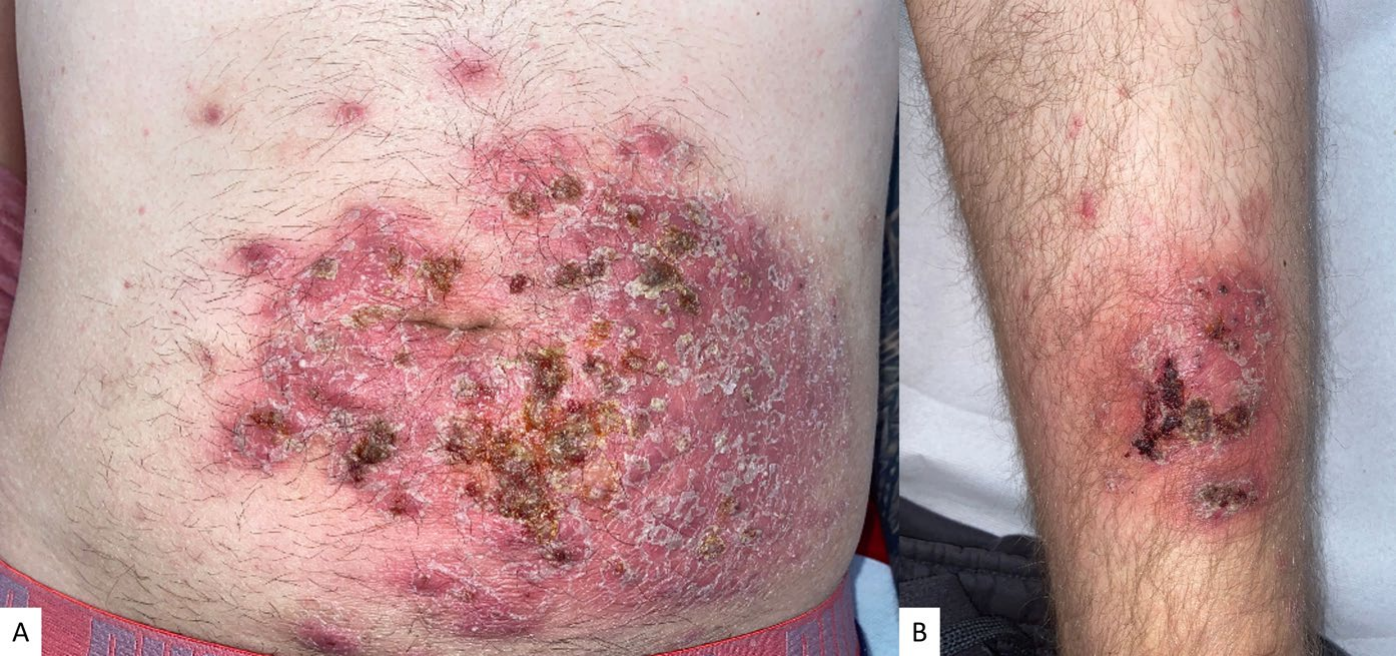
Well-demarcated erythematous, indurated plaques with yellow crusting and pustules were observed on the lower left abdomen (**A**) and left distal thigh (**B**)

**Fig. 2:**
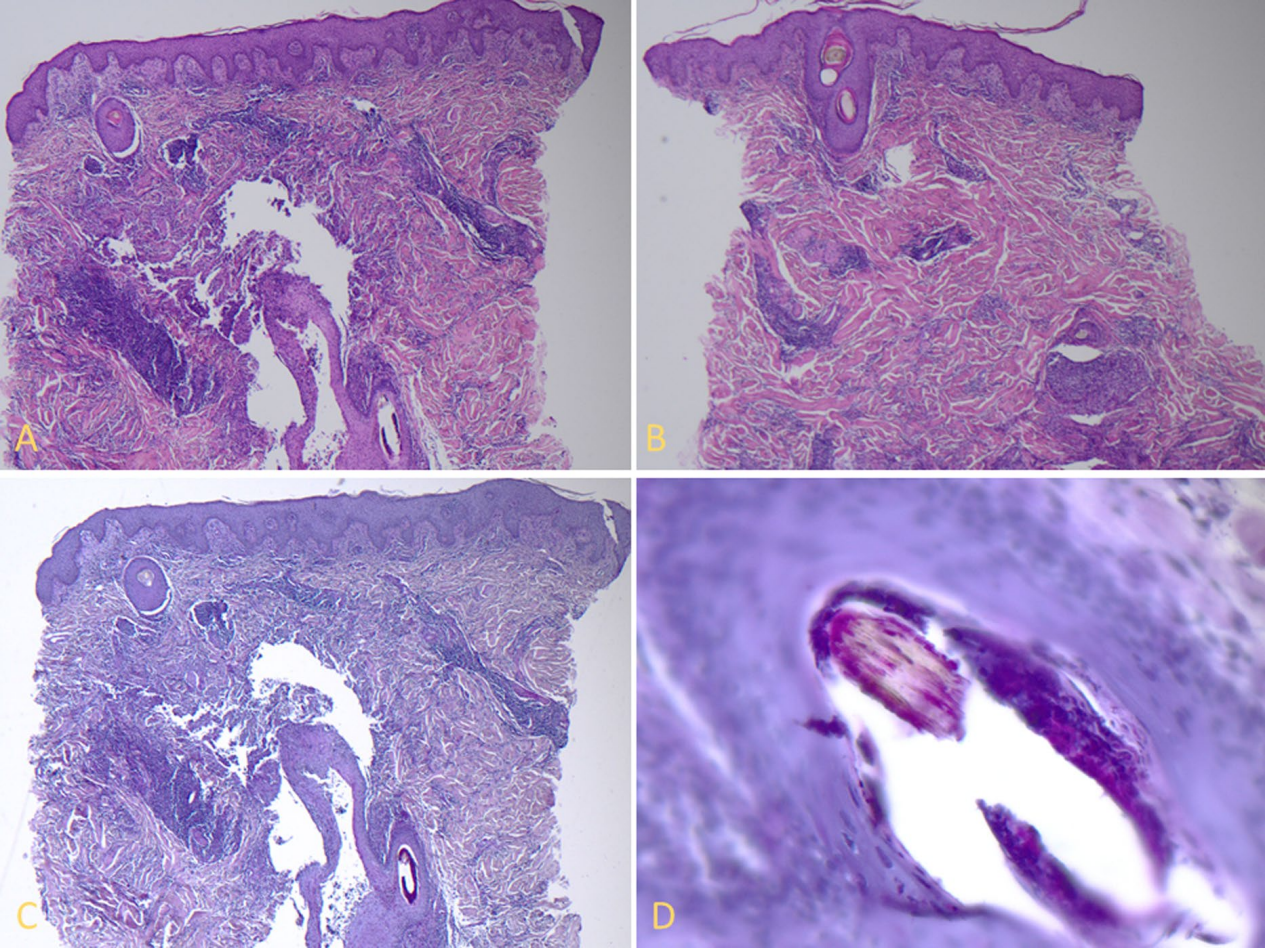
**A**–**B** A 5 mm punch biopsy shows a regularly structured epidermis with intraepidermal corkscrew hairs. The reticular dermis shows a deep perifollicular accentuated mixed infiltrate (H&E staining; 125× magnification). **C**–**D** The same biopsy, highlighted with PAS staining, shows fungal elements surrounding and invading a partially destroyed hair shaft (**C** and **D**: PAS staining; **C**: 125× magnification; **D**: 400× magnification)

During the examination, well-demarcated erythematous, indurated plaques with yellow crusting and pustules were observed on the lower left abdomen (Fig. 1A) and left distal thigh (Fig. 1B). Large superficial scaling and perifocal erythematous nodules were also present. Blood tests revealed leukocytosis (16 × 10^9/L^, normal range 3.50–10.00 G/l) and elevated C-reactive protein (39 mg/l; normal range < 5 mg/l). Our initial differential diagnosis was superinfected bacterial folliculitis; however, the lack of improvement under systemic antibiotic therapy prompted us to further investigate the patient's history. Upon questioning, he mentioned owning a dog and a cat, the latter of which had changes in fur around the ears for at least two months. Therefore, we decided to perform a skin biopsy and deep swab tests. The skin biopsy revealed deep trichophytism (Fig. 2A–D), while deep swabs from pustules showed growth of Trichophyton mentagrophytes, a zoophilic dermatophyte often transmitted from animals to humans. The identification of Trichophyton mentagrophytes was confirmed using microscopic examination, followed by culture on Sabouraud Dextrose Agar.

**Fig. 3:**
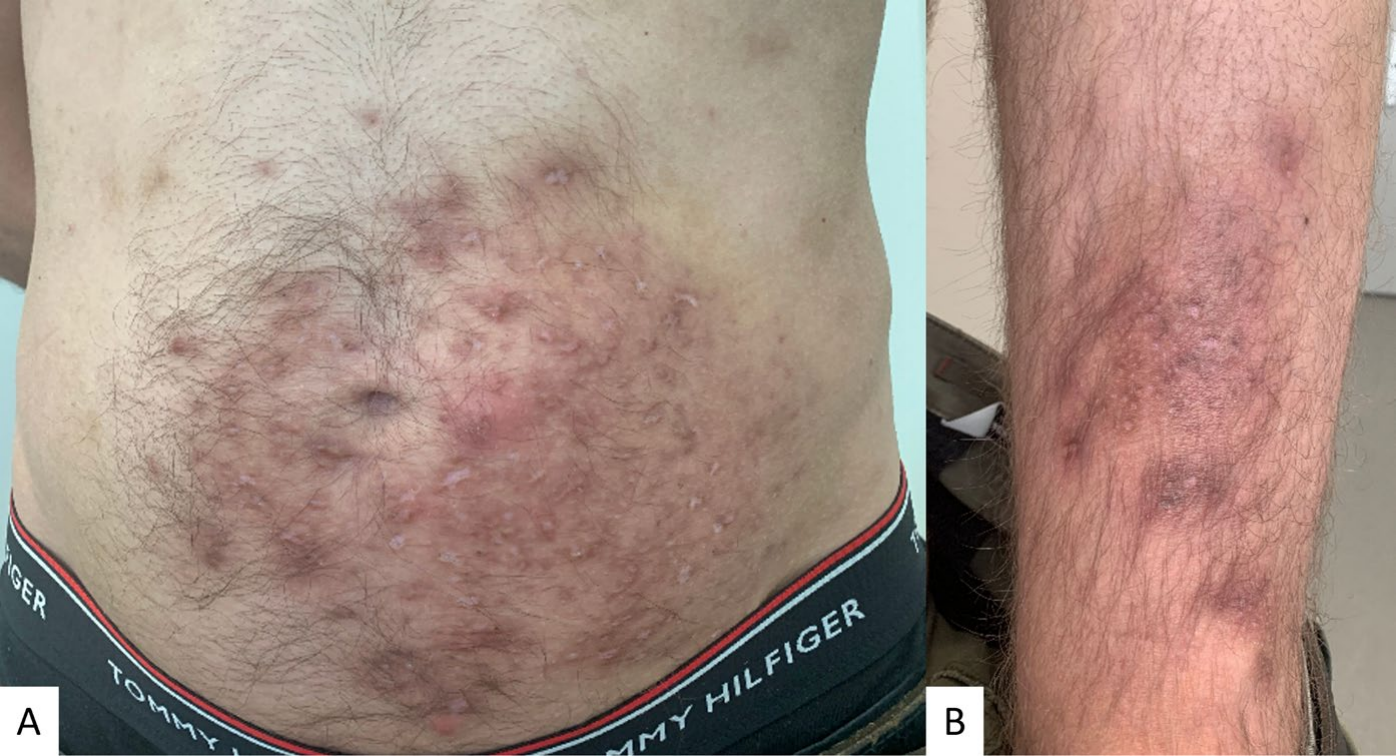
Post-inflammatory hyperpigmentation on the lower left abdomen (**A**) and left distal thigh (**B**) 8 weeks after the oral and topical treatment

Consequently, we initiated treatment with Terbinafine 250 mg once daily for 8 weeks, along with topical Econazole twice daily for a total of 8 weeks, which resulted in significant clinical improvement (Fig. 3 A–B).

This case underscores the importance of considering fungal infection as a differential diagnosis in patients presenting with lesions resembling superinfected folliculitis, in order to avoid delays in initiating appropriate treatment.

